# Effects of Biological and Chemical Degradation on the Properties of Scots Pine Wood—Part I: Chemical Composition and Microstructure of the Cell Wall

**DOI:** 10.3390/ma15072348

**Published:** 2022-03-22

**Authors:** Magdalena Broda, Carmen-Mihaela Popescu, Simon F. Curling, Daniel Ilie Timpu, Graham A. Ormondroyd

**Affiliations:** 1 Department of Wood Science and Thermal Techniques, Faculty of Forestry and Wood Technology, Poznań University of Life Sciences, ul. Wojska Polskiego 38/42, 60-637 Poznan, Poland; 2 Petru Poni Institute of Macromolecular Chemistry of the Romanian Academy, 41A Grigore Ghica Voda Alley, 700487 Iasi, Romania; mihapop@icmpp.ro (C.-M.P.); dtimpu@icmpp.ro (D.I.T.); 3 Centre of Wood Science and Technology, Edinburgh Napier University, 37 Bankhead Crossway South, Edinburgh EH14EP, UK; 4 BioComposites Centre, Bangor University, Deiniol Road, Bangor LL57 2UW, Gwynedd, UK; s.curling@bangor.ac.uk (S.F.C.); g.ormondroyd@bangor.ac.uk (G.A.O.)

**Keywords:** degraded wood, decayed wood, wood microstructure, surface area, porosity, cellulose crystallinity, chemical degradation, decay, wood polymers, wood properties

## Abstract

Research on new conservation treatment for archaeological wood requires large amounts of wooden material. For this purpose, artificial wood degradation (biological—using brown-rot fungus *Coniophora puteana*, and chemical—using NaOH solution) under laboratory conditions was conducted to obtain an abundance of similar samples that mimic naturally degraded wood and can serve for comparative studies. However, knowledge about its properties is necessary to use this material for further study. In this study, the chemical composition and microstructure of degraded cell walls were investigated using FT-IR, XRD, helium pycnometry and nitrogen absorption methods. The results show that biological degradation caused the loss of hemicelluloses and celluloses, including the reduction in cellulose crystallinity, and led to lignin modification, while chemical degradation mainly depleted the amount of hemicelluloses and lignin, but also affected crystalline cellulose. These changes affected the cell wall microstructure, increasing both surface area and total pore volume. However, the chemical degradation produced a greater number of mesopores of smaller size compared to fungal decomposition. Both degradation processes weakened the cell wall’s mechanical strength, resulting in high shrinkage of degraded wood during air-drying. The results of the study suggest that degraded wood obtained under laboratory conditions can be a useful material for studies on new consolidants for archaeological wood.

## 1. Introduction

Wood is one of the most universally used natural materials that humans have employed for many different applications, including construction, weapons, transportation, furniture, art, and fuel [1]. Every year, numerous old wooden objects are discovered worldwide that constitute priceless evidence of the development of our civilisation. However, since wood, as is the case with all biomaterials, is susceptible to natural degradation processes, wooden historical artefacts need not only careful conservation to preserve the material that has survived, but also effective protection against further degradation to save the wooden cultural heritage for future generations [2,3,4].

Research on new conservation agents and preservatives requires large amounts of wood material of similar structure and properties to test the effectiveness of different chemicals and investigate the performance of treated samples. Unfortunately, it is hard to obtain enough historical wood for this purpose—one cannot afford to sacrifice valuable artefacts for experiments. Moreover, such wood is usually unevenly degraded, which makes it extremely difficult to source a sufficient number of samples of similar properties for research. Hence, the idea for artificial wood degradation under laboratory conditions using the alkali treatment and one of the wood-degrading fungi to mimic degraded archaeological wood and obtain larger amounts of similarly degraded wood specimens. 

Alkaline hydrolysis using NaOH solution is commonly applied in industry for delignification of wood (e.g., in paper production) or other lignocellulosic biomass (to obtain natural fibres with improved surface adhesion used for the production of fibre-reinforced composites) [5,6,7,8]. Although the process effectively degrades and dissolves lignin (up to 90–95% of its content in wood), it is not fully selective and can also decompose wood carbohydrates, mainly hemicelluloses, and affect cellulose crystallinity [8,9,10]. Chemical degradation with sodium hydroxide has already been used before in research on new conservation agents to mimic deteriorated wood [11,12,13].

Biological degradation occurs naturally as part of matter cycles in the environment. Depending on the conditions (including moisture content, temperature, oxygen availability), wood can be decomposed by different organisms. In terrestrial environments, the most common wood degraders are wood-decaying fungi: brown-rot, white-rot and soft-rot. They mainly degrade wood carbohydrates but can also decompose lignin (particularly white-rot fungi) [14,15]. In aquatic ecosystems, wood is usually decomposed by soft-rot fungi and bacteria (erosion, tunnelling and cavitation bacteria), which mainly decompose polysaccharides, but tunnelling bacteria can also degrade lignin-rich regions of the cell wall [16,17,18]. In this research, brown-rot fungus *Coniophora puteana* (Shum.: Fr.) P. Karst was chosen for wood degradation since it can effectively decompose softwood (pine was used in the study), the control of fungal activity under laboratory conditions is relatively easy and the protocols for mycological tests are well-established [19].

Degradation processes change the content and structure of particular wood polymers (cellulose, hemicelluloses and lignin) in the cell wall and interfere with their mutual interactions and arrangement. As a result, all the wood properties change compared to sound wood [20,21,22,23]. 

To study and understand the effect of different conservation agents on degraded wood, complete knowledge about the properties of wood itself is necessary. Therefore, the first step of the research was to recognise the effect of biological and chemical degradation on wood. This paper focuses on the changes in the chemical composition and microstructure of the degraded wood cell wall. Additionally, physical properties of degraded wood such as mass loss, density and shrinkage during drying are discussed. Wood chemical composition was studied using Fourier-Transform Infrared Spectroscopy (FT-IR), and cellulose crystallinity was evaluated based on the X-ray diffraction (XRD) analysis. The cell wall microstructure was analysed using a nitrogen absorption method and a helium pycnometry that allow for measuring the cell wall pore volume, surface area and density, respectively. All these changes strongly affect the moisture and mechanical properties of the degraded wood, which will be presented in the next paper (Part II) to provide complete characteristics of the artificially degraded wood. That knowledge will help better understand the relationships between changes in wood chemical composition and the selected properties resulting from different types of degradation. This is necessary for further research on new conservation agents for waterlogged archaeological wood. Furthermore, it will facilitate the development of new lignocellulosic biomass-based materials and preservatives to protect them against degradation.

## 2. Materials and Methods

### 2.1. Materials

Contemporary Scots pine (*Pinus sylvestris* L.) sapwood sourced from commercial timber merchants in the Wielkopolska Region, Poland, was used as research material. The main object of the presented study was chemically and biologically degraded pinewood prepared under laboratory conditions (see Section 2.2.1. Sample preparation) to mimic waterlogged archaeological wood and serve for further research. In addition, sound wood was used as a reference.

### 2.2. Methods

#### 2.2.1. Sample Preparation—Wood Degradation

To mimic degraded archaeological wood, sound pinewood blocks with dimensions of 20 mm × 20 mm × 10 mm (in radial, tangential and longitudinal direction) were subjected to chemical and biological degradation.

Biological degradation was performed using *Coniophora puteana* (Shum.: Fr.) P. Karst BAM 112 (BAM Ebw. 15). Wood specimens were sterilised by gamma irradiation (2.5 Mrad) and installed in sterile Kole flasks on glass supports placed on 5% malt agar pre-inoculated 1 week earlier. The flasks were plugged with cotton plugs and incubated in a growing chamber at 24 ± 1 °C and 70 ± 5% relative humidity. After 8 weeks, the samples were taken out from the flasks and the mycelium brushed off. To calculate average wood mass loss upon degradation, 40 samples were oven-dried at 105 °C to constant weight (about 24 h) and weighed. The average mass loss expressed as a percentage of the initial oven-dry mass of the sample was 39 ± 5%. The remaining samples were immersed in distilled water immediately after brushing to keep them filled with water, thus mimicking waterlogged wood required in further research.

Chemical degradation was performed using 50% NaOH. Samples were immersed in an alkaline solution for 3 weeks, then thoroughly washed with distilled water until neutral pH. Forty specimens were then oven-dried at 105 °C to constant weight (about 24 h) and weighed. The average mass loss of degraded samples was 17 ± 1%. Similarly to biologically degraded ones, the remaining samples were left in distilled water for further study.

The bulk density of sound and degraded wood (ρ) was calculated as the ratio of the sample weight to its volume after air-drying and conditioning under the temperature of about 20 °C and relative humidity of about 50%.

#### 2.2.2. Infrared Spectroscopy

The chemical composition of wood was characterised using the Fourier-Transform Infrared Spectroscopy (FT-IR) technique [24,25,26]. Five replicates for each wood type were air-dried and conditioned at 21 ± 1 °C and 40 ± 5% relative humidity until equilibrium moisture content was achieved. After that, the samples were powdered and sieved, and the powder fraction particles with a diameter of <0.2 mm was further used. Infrared spectra were recorded in the 4000–400 cm^−1^ region, using a Bruker ALPHA FT-IR spectrometer (Bruker, Billerica, MA, USA) in KBr pellets (2 mg wood powder/200 mg KBr) with 4 cm^−1^ resolution. Five spectra for each wood specimen were recorded, averaged and processed using Grams 9.1 software (Thermo Fisher Scientific, Waltham, MA, USA). For principal component analysis (PCA), the 1800–1550 cm^−1^ region from the FT-IR spectra was used. The processing was carried out in the Origin 2021 program (OriginLab Corporation, Northampton, MA, USA).

#### 2.2.3. X-ray Diffraction

X-ray diffraction analysis (XRD) was used as a technique supplementary to FT-IR to evaluate the cellulose crystallinity in wood degraded with different methods [24,25,27]. A Diffractometer D8 ADVANCE (Bruker AXS, Karlsruhe, Germany) with Cu-K radiation (λ = 0.1542 nm), a parallel beam with Gobel mirror and a Dynamic Scintillation detector was employed for the measurements. The measurement conditions were 40 kV, 30 mA, 2 s/step, and 0.02 degree/step. The diffractograms obtained were processed using Grams 9.1 software (Thermo Fisher Scientific, Waltham, MA, USA). The Voight profile was applied for the amorphous background, while mixed Gaussian-Lorentzian profiles were used for the crystalline signals. The crystallinity degree was calculated using Hermans and Weidinger’s equation [27]:

(1) $$\mathrm{Cr}.I.\%=\frac{A_{\mathrm{cr}}}{A_{t}}\times 100$$

where Cr.I. % is the crystallinity degree, A_cr_ is the sum of the crystalline signals and A_t_ is the total area under diffractogram.

#### 2.2.4. Surface Area and Pore Volume Measurements

Alterations in the wood cell wall microstructure after biological and chemical degradation were investigated using a nitrogen absorption method [28,29,30]. Measurements were performed on a Gemini Surface Area Analyser (Micromeritics Instrument Corporation, Norcross, GA, USA) using de-gassed wood specimens. Five nitrogen sorption isotherms were recorded at liquid nitrogen temperature for each wood type. The surface area of the cell wall was calculated based on the Brunauer–Emmet–Teller (BET) theory [31] that assumes that the nitrogen gas has access to the entire cell wall surface, and the surface area can be then calculated from the volume of nitrogen adsorbed on the cell wall at different partial pressures. Wood porosity (i.e., total pore volume and their size distribution) was analysed using the Barrett–Joyner–Halenda (BJH) method [32]. Although the method is not appropriate for measurements of micro- and mesopores with a diameter below 4 nm, we have already shown that it is suitable for making comparisons between analysed samples [29,30]. Micromeritics Stardriver software was employed for all calculations.

#### 2.2.5. Helium Pycnometry

Helium pycnometry is applied to measure wood porosity and determine the cell wall density [33,34,35]. By measuring the difference in the volume of helium injected to a certain pressure into an empty measurement chamber and chamber filled with a sample, the method allows for accurate measurement of a sample’s density when its mass is known [35]. 

The cell wall density of sound and degraded wood was determined with the Accupyc 1330 Micromeritics (Micromeritics Instrument Corporation, Norcross, GA, USA) gas pycnometer using helium gas 99.99% pure. Wood samples with dimensions fitted to the pycnometer chamber (10 cm^3^) were oven-dried for 12 h at 104 °C and cooled in a desiccator; then, they were weighed, inserted in the pycnometer and measured. Before the measurements, the pycnometer was calibrated using certified calibration spheres with a volume of 6.371684 cm^3^ (Micromeritics Instrument Corporation, Norcross, GA, USA).

#### 2.2.6. Wood Shrinkage upon Drying and Density of Dried Wood

One of the main problems with degraded waterlogged wood is its dimensional instability upon drying, leading to distortions, cracking, and even complete destruction of a wooden artefact [3]. Applying proper wood consolidants can prevent the wood from shrinking and cracking. However, knowledge about the shrinkage of untreated wood is necessary to evaluate the stabilising effect of new consolidants. Therefore, based on the measurements of sample dimensions before (at a wet/waterlogged state) and after air-drying, volumetric shrinkage (S) for biologically and chemically degraded wood was calculated according to Equation (2):

(2) $$S=\frac{V_{0}-V_{1}}{V_{0}}\times 100$$

where V_0_ is the initial volume of the sample (in the waterlogged state) and V_1_ is the final volume of the dried sample.

## 3. Results and Discussion

### 3.1. Fourier-Transform Infrared Spectroscopy (FT-IR)

Infrared spectroscopy was involved in evaluating the modifications that occurred in the wood after 8 weeks of exposure to brown-rot fungi *C. puteana* and after chemical treatment (alkali treatment). Both spectra and their second derivatives highlighted significant differences compared to the control sample spectrum—differences associated with chemical alterations of wood polymers.

The first region (left side of the break—3700–2700 cm^−1^) is assigned to stretching vibrations of different inter- and intra-molecular hydrogen bonds, as well as symmetric and asymmetric stretching vibrations of methyl and methylene groups [24,36,37]. 

The main differences in this region are observed for the band from 3420 cm^−1^ in CP (assigned to O(2)H…O(6) intra-molecular hydrogen bonds in the crystalline regions of cellulose [24,36,37]) that is shifted to 3417 cm^−1^ in both, biodegraded and chemically treated pine wood samples. In ChP, this band also increases in intensity compared to CP. 

**Figure 1:**
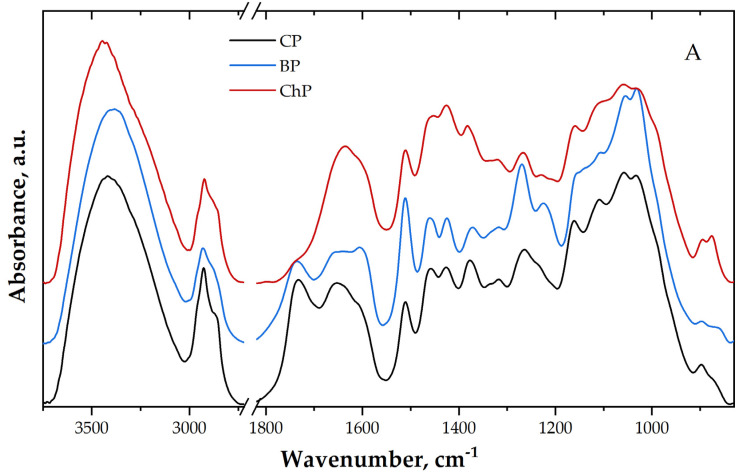

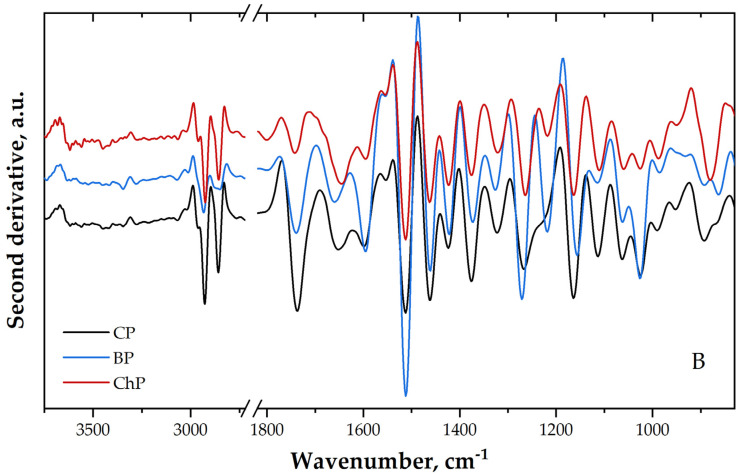
Infrared spectra (**A**) and their second derivatives (**B**); CP—contemporary sound pine, BP—biologically degraded pine, ChP—chemically degraded pine.

From Figure 1B, it can be seen that the band from 3344 cm^−1^ (assigned to O(3)H…O(5) intra-molecular H bonds in cellulose [24,36,37]) is shifted to 3347 cm^−1^ and increases in intensity in BP, and to 3340 cm^−1^ and decreases in intensity in ChP compared to CP spectrum. The band from 3276 cm^−1^ (assigned to O(6)H…O(3) intermolecular hydrogen bonds in crystalline cellulose allomorph monoclinic phase Iβ [24,36,37]) presents similar behaviour to the previously mentioned band—it increases in intensity and is located at 3278 cm^−1^ in BP spectrum and decreases in intensity and is shifted to 3272 cm^−1^ in the ChP spectrum. The band from 3212 cm^−1^ in CP spectrum (assigned to O(6)H…O(3) intermolecular hydrogen bonds in crystalline cellulose allomorph triclinic phase Iα [24,36,37]) is shifted to 3223 cm^−1^ and decreases in intensity in BP spectrum.

BP spectrum indicates differences also for the bands assigned to stretching vibration of methyl and methylene groups from 2934, 2871 and 2850 cm^−1^, compared to the other two samples spectra. These bands decrease in intensity and are shifted to a different wavenumber. This modification might indicate the degradation with release of low molecular compounds from amorphous carbohydrates, but also lignin demethoxylation caused by brown-rot [38,39].

The increase in the intensity of the bands associated with intra- and inter-molecular hydrogen bonds in crystalline cellulose allomorph monoclinic phase Iβ indicates an increment of this phase moiety in biodegraded pine wood, and the decrease of the bands associated with inter-molecular H bonds in the crystalline cellulose allomorph triclinic phase Iα indicates a reduction in this phase moiety in biodegraded pine wood. This further indicates the degradation of amorphous carbohydrates during the biodegradation process. On the other hand, a decrease in intensity of these bands in ChP spectrum may suggest degradation of crystalline domains of cellulose as well.

Compared to sound pine wood (CP), the spectrum of ChP sample presents the main differences in the 1800–1550 cm^−1^ region. The bands from 1742 and 1657 cm^−1^ (assigned to C=O stretching vibration of carboxyl and acetyl groups and to conjugated C–O stretching vibration in quinones [24,37]) decreases drastically in intensity for this sample, while the band from 1639 cm^−1^ (assigned to absorbed O–H [24,37]) shows a slight increase in intensity compared to CP sample. The 1742 cm^−1^ band’s intensity decrease indicates a reduction in the amount of carbonyl and carboxyl groups, further indicating the degradation of hemicelluloses and lignin after the treatment, characteristic for alkali-treated wood [8,9,10].

In the BP spectrum, a slight decrease in the intensity of the band from 1741 cm^−1^ and an increase in the intensity of the band from 1592 cm^−1^ (assigned to C=C stretching mode of aromatic skeletal in lignin [24,37]) can be observed. At the same time, the band from 1638 cm^−1^ appears to be more similar to a shoulder (similarly to CP) compared to ChP. These results are in line with the knowledge that the brown-rot fungi induce a reduction in the amount of hemicelluloses and, at the same time, an increase in the value of aromatic lignin moieties [15,40].

Furthermore, in the fingerprint region, BP spectrum present differences as follows: the bands from 1512, 1462, 1423, 1270, 1060 and 1027 cm^−1^ (assigned to aromatic C=C stretching vibration in lignin structure, C–H deformation vibration in lignin and carbohydrates, C−H bending mode in cellulose and C–O stretching vibration in lignin, C−O stretching mainly from C(3)−O(3)H in cellulose I and to C−O and C−C stretching ring vibration in carbohydrates [24,37]) increase in intensity in BP spectrum compared to CP spectrum, while a decrease in intensity for the bands from 1374, 1322, 1160, 1111 cm^−1^ (assigned to C–H deformation vibration in carbohydrates, C–H stretching vibration in cellulose and C_l_–O stretching vibration in syringyl derivatives, C–O–C stretching vibration in cellulose (in crystalline regions) and C–O stretching vibration [24,37]) can be seen compared to CP spectrum. The band from 1220 cm^−1^ increases in intensity, and it is a combination of the two bands from 1232 and 1215 cm^−1^ (assigned to C−O−C stretching mode of the pyranose ring and C=O stretching vibration in lignin and xylan [24,37]) (observed in CP spectrum). These modifications indicate alterations in wood structure characteristic for biodegradation by the fungus *C. puteana* the samples were exposed to [40,41]. 

The chemically treated sample (ChP) spectrum presents an intensity increase in the bands from 1268 cm^−1^ (assigned to C−H bending mode in cellulose and C–O stretching vibration in lignin [24,37]), and an intensity decrease in the bands from 1464, 1424, 1378, 1317, 1063 and 1023 cm^−1^ (assigned to C–H deformation vibration in lignin and carbohydrates, C–H deformation vibration in carbohydrates, CH_2_ rocking vibration in cellulose, C−O stretching vibration mainly from C(3)−O(3)H in cellulose I and C−O and C−C stretching ring vibration in carbohydrates [24,37]). The bands from 1227 and 1211 cm^−1^ are shifted to lower wavenumber compared to CP spectrum (1232 and 1215 cm^−1^) and are decreased in intensity, while the band from 1117 cm^−1^ is shifted to higher wavenumber (compared to CP spectrum, at 1113 cm^−1^) and is also decreased in intensity. These bands are assigned to C−O−C stretching mode of the pyranose ring, C=O stretching vibration in lignin and xylan and C−O stretching vibration [24,37]. The observed changes in the ChP spectrum indicate the reduction in the lignin and hemicelluloses amount in comparison with CP sample.

**Figure 2:**
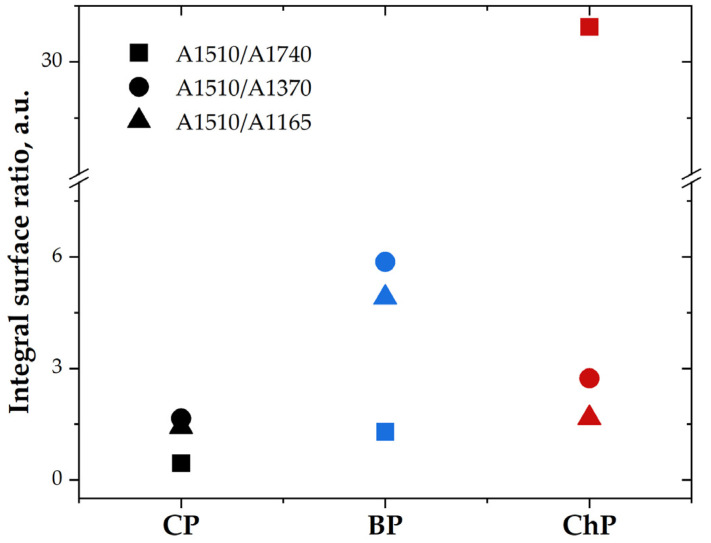
The integral surface ratio of 1510 cm^−1^ band versus 1740, 1370 and 1165 cm^−1^ bands; CP—contemporary sound pine, BP—biologically degraded pine, ChP—chemically degraded pine.

To highlight the modifications in the lignin and carbohydrate amounts caused by biodegradation and chemical treatment compared to control sound wood, the integral area ratios of the lignin band from 1510 cm^−1^ against the carbohydrates bands from 1740, 1370 and 1165 cm^−1^ (assigned to C=O stretching vibration of carboxyl and acetyl groups attributed to the presence of hemicelluloses and lignin, C–H deformation vibration in carbohydrates, C–O–C stretching vibration in cellulose, especially in crystalline regions [24,37]) were calculated (Figure 2).

The A1510/A1740 ratio presents a slightly increased value for BP, compared to CP, while the value for ChP is very high. This indicates the elimination of hemicelluloses and lignin from the chemically treated pine wood sample.

The A1510/A1370 and A1510/A1165 ratios present the highest values for the biodegraded sample (BP), and only slightly higher value for the ChP compared to CP. The increased values of these ratios in BP indicate the selective degradation of carbohydrates and a reduction in the crystalline regions during the biodegradation process with brown-rot fungi *C. puteana*. It is known that *C. puteana* induces the loss of carbohydrates from the wood structure, and similar results were observed by Durmaz and co-workers in their study [41]. On the other hand, cellulose crystallinity (based on the A1510/A1165 ratio) in alkali-treated wood was found slightly higher than the control sample, indicating a partial reduction in amorphous cellulose [9]. The higher A1510/A1370 ratio (compared to CP) points to the reduction in wood carbohydrates. These results confirm that hemicelluloses and lignin were partially removed or modified in chemically degraded wood [7,9].

### 3.2. Principal Component Analysis (PCA)

Principal component analysis (PCA) was used to extract information regarding the differences that may appear between the studied samples [24,42]. 

**Figure 3:**
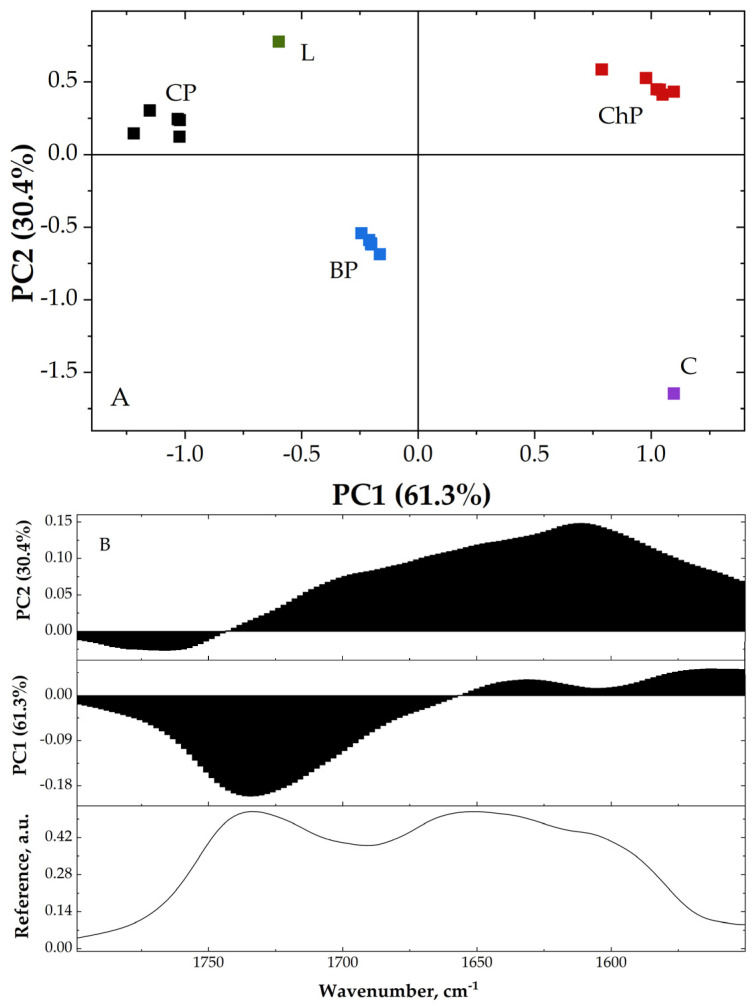
PC scores plot (**A**) and loadings plot (**B**); L—lignin, C—cellulose, CP—contemporary sound pine, BP—biologically degraded pine, ChP—chemically degraded pine.

For PCA analysis, the region between 1800 cm^−1^ and 1550 cm^−1^ was used. This region presents the highest differences between the samples. Moreover, to highlight the similarities and differences, aside from the control sample spectra, we also used the spectrum of thermo-mechanically bleached pulp and lignin (extracted from softwoods). PC scores and loadings were plotted and are presented in Figure 3.

From Figure 3A, one can observe that the PC1 (principal component 1) describes 61.3% and PC2 (principal component 2) 30.4% of data variance; therefore, 91.7% of the existed variances from all spectra can be captured using only these two dimensions.

The control pine wood (CP) presents negative PC1 values, but positive and close to zero PC2 values, BP presents negative values for both PC scores (although the PC1 values are closer to 0), while ChP presents positive values for both PC1 and PC2 scores. 

The L present negative PC1 values and positive PC2 values, while C presents positive PC1 and negative PC2 values.

As can be observed, PC1 describes the similarities between the carbohydrates/lignin content in the samples. CP, BP and L present negative values, while ChP and C present positive PC1 values. The scores present negative relationship between carbohydrates and lignin.

The loading plots (Figure 3B) indicate the variation of the bands, highlighting the chemical composition differences of the studied wood samples.

### 3.3. X-ray Diffraction (XRD)

Generally, X-ray diffraction is used to determine the crystallinity degree of wood and identify the modifications in crystallinity degree during different treatments or the degradation processes. From all wood components, only cellulose is partially crystalline, and the pattern of an X-ray diffractogram presents the crystalline bands at about 22−22.5° 2θ assigned to 200 crystallographic plane of cellulose I, about 14.6–15.5° 2θ assigned to 101 crystallographic plane of cellulose I, about 16.5−17.0° 2θ assigned to the 10-1 crystallographic plane of cellulose I, about 20.0–21.0° 2θ assigned to 102 crystallographic plane of cellulose I, and at about 18.5–19.2° 2θ, a background assigned to amorphous part of cellulose with contributions from lignin and hemicelluloses [24,43].

**Figure 4:**
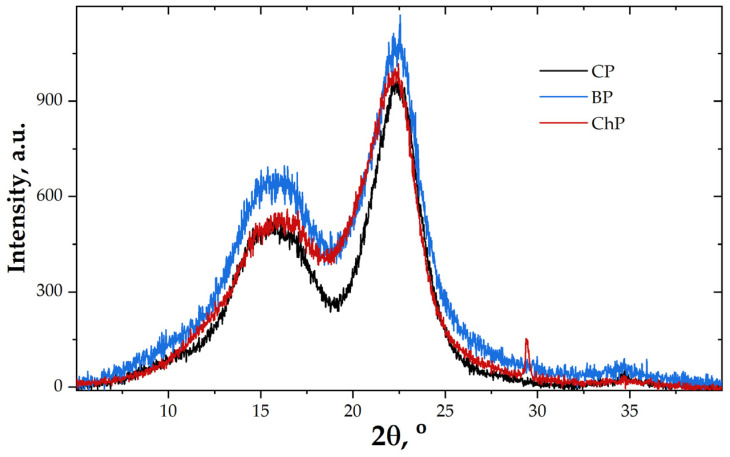
X-ray diffractograms of pine wood samples; CP—contemporary sound pine, BP—biologically degraded pine, ChP—chemically degraded pine.

The CP sample diffractogram (Figure 4) indicates a typical wood pattern, with the bands from 15.0 and 16.9 merged in one band with a maximum at about 15.5° 2θ, while the small band from 20.8 is merged with the band from 22.5° 2θ. The crystallinity degree was found to be of 55.9%.

The BP diffractogram shows a higher amorphous background and reduced signal of the bands from 22.5° 2θ. The crystallinity degree of this sample was found to be of 50.1%. The results confirm the reduction in crystalline cellulose caused by brown-rot fungus.

In the ChP diffractogram, a small shoulder at about 12.0° 2θ appears, an increased signal at 16.4° 2θ can be seen, and the band assigned to 200 crystallographic planes of cellulose I is shifted to lower 2θ values. All these modifications indicate the presence of cellulose II aside from cellulose I after the chemical (alkali) treatment, which is characteristic of this type of degradation [7].

Furthermore, the decrease in the crystallinity degree of this sample from 55.9% (in CP) to 52.7% (in ChP) indicates that the treatment did not remove only the amorphous polymers from the wood structure, but also must have affected the crystalline regions. Usually, NaOH treatment causes an increase in relative crystallinity due to removal of lignin and hemicelluloses [7,8,9].

### 3.4. Wood Shrinkage and the Cell Wall Characteristics

Biological and chemical degradation, resulting in the wood mass loss due to decomposition of its particular polymers, weakened the cell walls, making the samples more susceptible to shrinkage upon air-drying. For chemically degraded pine (ChP), shrinkage was about 25%, while for biologically degraded wood (BP), it was about 22% (Table 1). Considering the significant difference in the mass loss between ChP and BP (about 17% vs. over 38%, Table 1), the similar shrinkage level may seem surprising. However, apparently, not the mass loss itself but the specific changes in chemical composition and the resulting microstructure and mechanical strength of the cell walls play a more significant role in the process [44,45].

The cell wall density was similar for all samples, despite the degree of degradation and the higher wood bulk density for chemically treated wood, presumably caused by the collapse of the degraded cell wall during air-drying (Table 1).

**Figure 5:**
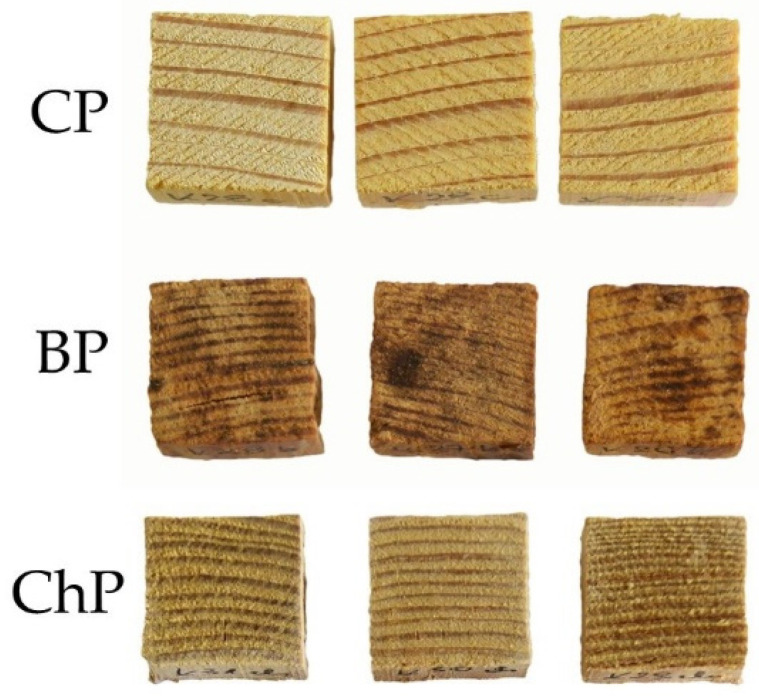
Air-dried pine samples: CP—sound wood, BP—biologically degraded wood, ChP—chemically degraded.

Different degradation processes also changed the appearance of the wood (Figure 5). Visually, the colour of dry chemically degraded wood was similar to undegraded wood, which was only slightly faded due to the lignin degradation [46], with small cracks visible on the surface of most of the specimens. Low in hemicelluloses and cellulose, biologically degraded wood was darker and more brownish, which was a result of the higher relative content of partially oxidized lignin [15,47].

Degradation also affected the microstructure of the cell walls. It is visible in the approximately twofold increase in the total pore volume and surface area for degraded wood compared with undegraded (Table 1), despite the shrinkage and presumed collapse of the degraded cell walls during drying that undoubtedly reduced both their surface area and pore volume [30]. 

An increase in surface area was due to an increase in the number and size of pores within the wood structure due to the degradation of the wood cell wall polymers. Alkaline treatment, as confirmed by FT-IR and XRD analyses, partially removed hemicelluloses and lignin and also affected cellulose. The process involves high cellulose swelling and changes in its structure, resulting in the cell wall collapse during drying. These phenomena increase the number of pores in the cell wall and its surface area [7,8]. The fungal attack degraded mainly hemicelluloses and cellulose and also affected lignin. In brown-rot decay, non-enzymatic chelator-mediated Fenton reactions cause depolymerisation of the lignin, allowing an attack on polysaccharides [40,48]. However, the lignin then repolymerises into a modified form separate from the cellulose. In this way, the pore structure could be changed.

**Figure 6:**
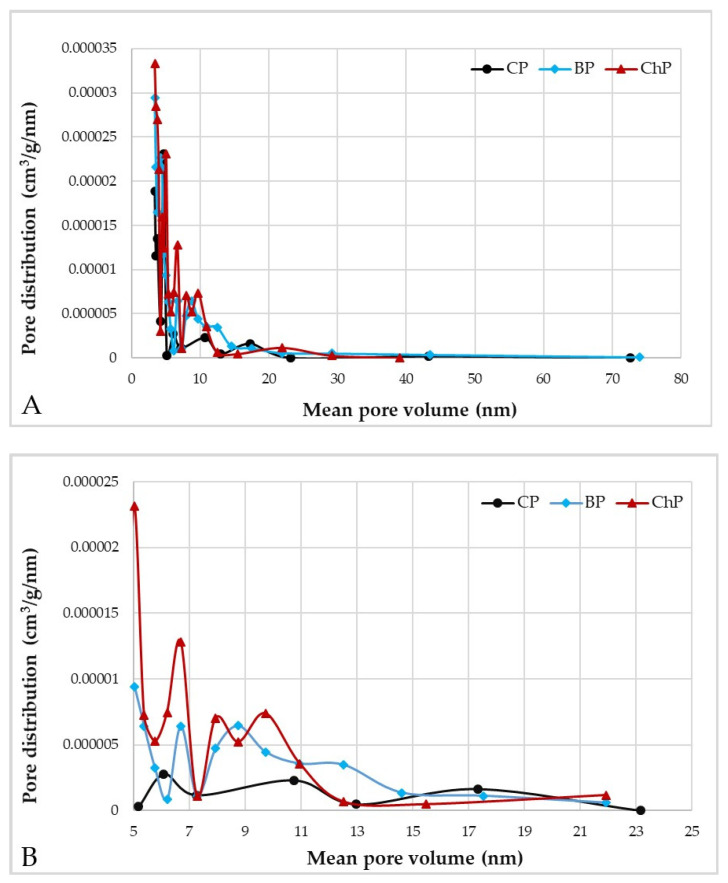
Pore distribution as a function of the pore volume illustrating the different distribution types for undegraded and artificially degraded pine wood (**A**) and a micro- and mesopore region showing more details of pore distribution (**B**); CP—contemporary sound pine, BP—biologically degraded pine, ChP—chemically degraded pine.

**Figure 7:**
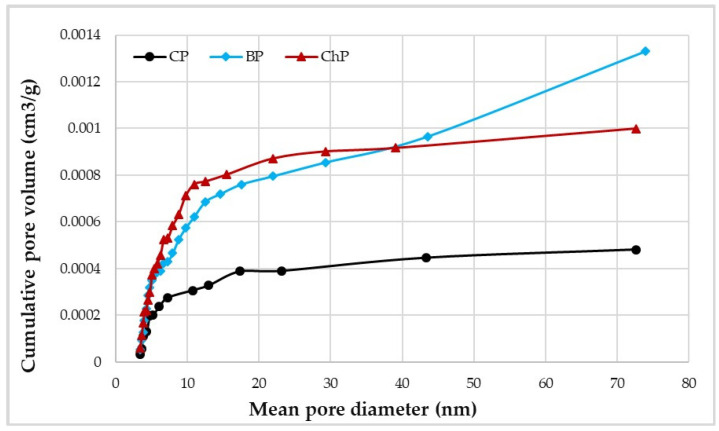
Cumulative pore volume as a function of the pore radius illustrating the different distribution types for undegraded and artificially degraded pine wood; CP—contemporary sound pine, BP—biologically degraded pine, ChP—chemically degraded pine.

The pore size and pore volume data (Table 1) show a clear difference in pore architecture for the degraded samples as opposed to the undegraded ones (Figure 6 and Figure 7). The data shown in Figure 6A shows little difference between degraded and sound wood for larger pore sizes, but at the smaller pore sizes, particularly within the 5–15 nm range, as shown in Figure 6B, clear evidence of more numerous pores can be noted. There are also differences between the effects of chemical and biological degradation. The chemical attack appears to produce more numerous small mesopores, presumably due to the collapse of the cell wall [7,8]. In comparison, the fungal attack also increases the amount of small mesopores in comparison to the undecayed wood, but also produces a few larger pores as well (Figure 7). Micropores below 4 nm in size (not measurable using nitrogen sorption) are also produced by brown-rot activity and this, combined with our experimental data, demonstrates that the fungal attack is more varied and complex than a single chemical attack, and thus may be more representative when attempting to replicate the condition of the archaeological specimens [49,50,51].

## 4. Conclusions

The results of the study clearly show that chemical and biological wood degradation under laboratory conditions significantly affect its chemical composition. Fungal degradation alters mainly wood polysaccharides, while alkali treatment degraded mainly hemicelluloses and lignin. From the conservation perspective, biologically degraded material seems then potentially more useful in further research on new conservation methods since its chemical composition is more similar to naturally decomposed archaeological wood, where mainly polysaccharide fraction is decomposed [24,52]. On the other hand, chemically degraded wood abundant in cellulose (which is a great source of nutrients for fungi) may be helpful in the studies on new antifungal treatments.

Regarding the cell wall microstructure changes, an increase in porosity of the cell wall due to the degradation of wood polymers improves its permeability and potentially facilitates further conservation treatment. On the other hand, it apparently weakens the mechanical strength of the cell wall, which is visible in the level of shrinkage of degraded wood after air-drying. This makes both materials potentially useful in the research on new consolidants that are supposed to reinforce the strength of decomposed wood.

The results or ongoing study concerning the other properties of biologically and chemically degraded wood will be presented in other papers to provide complete characteristics of the material.

## Figures and Tables

**Table 1:** Average values of mass loss, shrinkage, surface area, total pore volume, cell wall density and bulk density (ρ) for sound/undegraded (CP), biologically (BP) and chemically (ChP) degraded pine wood.

Wood Type	Mass Loss (%)	Shrinkage (%)	Surface Area (m^2^ g^−1^)	Total Pore Volume (cm^3^ g^−1^)	Cell Wall Density (g cm^−3^)	ρ (g cm^−3^)
CP	-	-	0.36 ± 0.02	0.0007	1.51 ± 0.002	0.44 ± 0.02
BP	38.5 ± 4.7	22.3 ± 4.6	0.70 ± 0.06	0.0015	1.54 ± 0.002	0.44 ± 0.03
ChP	16.8 ± 1.4	25.1 ± 4.2	0.79 ± 0.03	0.0012	1.57 ± 0.002	0.65 ± 0.02

## Data Availability

The data presented in this study are available on request from the corresponding author.
